# Automated quality control for genome wide association studies

**DOI:** 10.12688/f1000research.9271.1

**Published:** 2016-07-29

**Authors:** Sally R. Ellingson, David W. Fardo

**Affiliations:** 1 Division of Biomedical Informatics, College of Medicine, University of Kentucky, Lexington, KY, 40536, USA; 2 Cancer Research Informatics Shared Resource Facility, Markey Cancer Center, University of Kentucky, Lexington, KY, 40536, USA; 3 Department of Biostatistics,, University of Kentucky, Lexington, KY, 40536, USA

**Keywords:** Genome wide association, genotype information, quality control

## Abstract

This paper provides details on the necessary steps to assess and control data in genome wide association studies (GWAS) using genotype information on a large number of genetic markers for large number of individuals. Due to varied study designs and genotyping platforms between multiple sites/projects as well as potential genotyping errors, it is important to ensure high quality data. Scripts and directions are provided to facilitate others in this process.

## Introduction

Biases and errors can lead to erroneous associations in case-control association tests. Quality control (QC) that removes markers and individuals from a study that may introduce these biases can greatly increase the accuracy of findings. There are many examples of best practices for GWAS QC ^1, 2^. This paper describes some standard QC steps and also provides links to automated scripts to perform QC making the process easier and easily reproducible. Standard tools such as PLINK ^3^ and SMARTPCA ^4, 5^ are called by the scripts.

Due to the need for reproducibility in science, automated pipelines that can be used to repeat computational experiments and save relevant parameters is extremely important. Done step-by-step, the QC process can be quite lengthy (about 8 hours for an expert and almost certainly longer for a novice and/or someone with limited computational resources according to Anderson *et al.* ^1^) and difficult to repeat exactly. Here we present scripts that perform automated GWAS QC using a parameter file that can be saved to redo the process and save human time. A log file is produced that summarizes the process to easily compare different QC parameters and their effects on the data.

## Methods

### Implementation

**Figure 1.**
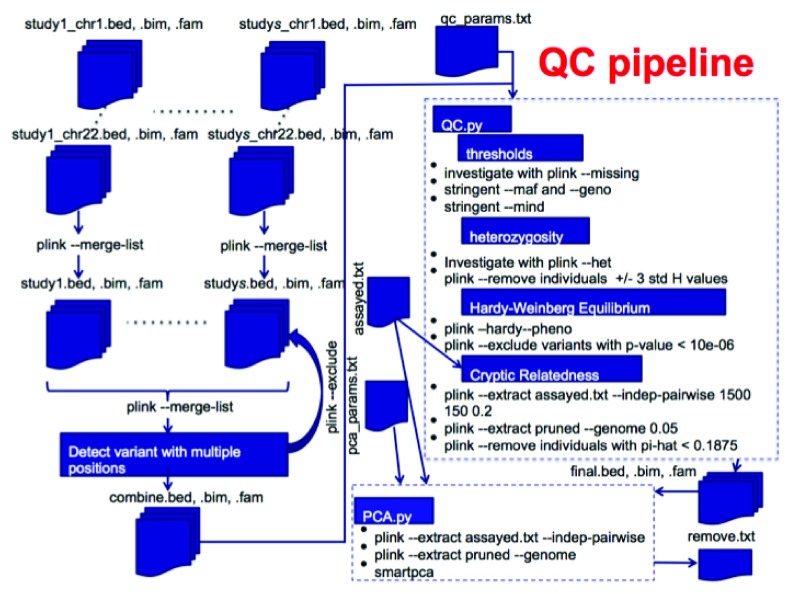
Full QC Pipeline.

*QC steps implemented in this pipeline.* The steps automated here mostly follow the notes on QC ^6^ developed by MikeWeale and also calls some R ^7^ scripts described in his notes during the QC pipeline. It is assumed that input files are already in PLINK format. Figure 1 shows a complete QC pipeline that includes combining data from multiple chromosomes and studies and two portions of the QC pipeline. There are two scripts, QC.py which takes advantage of PLINK calls and also PCA.py that does principal component analysis to investigate population stratification.

**Figure 2.**
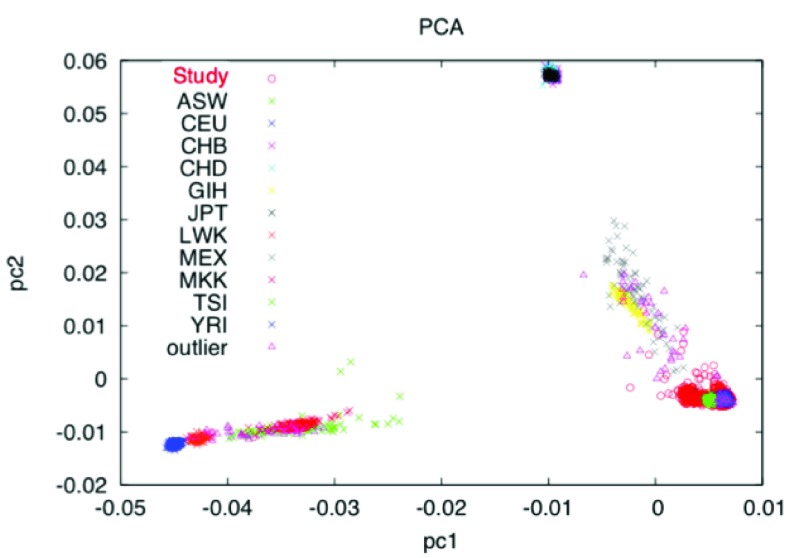
PCA with HapMap data included.

1. Gender mismatches
    The optional first step in the automated pipeline is a check for gender mismatch using the PLINK ‘-- check-sex’ command. This command compares the sex reported in the .fam file and the sex imputed from the X chromosome inbreeding coefficients. This step automatically removes individuals where problems are identified. The step was made optional because our dataset of interest is matched with phenotype/clinical data of higher accuracy. This step can be turned off using the parameter file as described below in the Operation section.
2. Thresholds
    The next steps in this pipeline include checking and applying thresholds for minor allele frequency (MAF), missingness for each individual, and missingness of markers. Minor allele frequency filtering is important because rare genotypes will not show up as often and thus will have less evidence in a GWAS and the calls will be less certain and it is also difficult to detect associations with them. Missingness can lead to false associations if it is non-random with respect to phenotypes or genotypes. Single nucleotide polymorphism (SNP) missingness is the complement to individual missingness and is correlated with SNP quality from the original genotyping assay. Missingness is investigated using PLINK ‘--missing’ and plots are generated as described by Weale ^6^. All the plots that are generated during the process are compressed at the end in order to facilitate downloading them when the process is performed remotely on a cluster.
    In order to attempt to retain the largest number of markers and individuals that pass QC there is an option to do a two-tiered missingness by individuals filtering. We noticed during testing that this could sometimes lead to final datasets with higher numbers of both. If a value is supplied to the #MIND1 parameter (described below in Operations), then this (expectedly non-stringent) threshold for PLINK ‘--mind’ is used first and the more stringent #MIND is applied in the same step as the PLINK ‘--geno’ and ‘--maf’ thresholds for missingness of markers and minor allele frequency, respectively. If a major reduction in the number of markers or individuals is found during these steps, investigation of the generated graphs can help adjust these thresholds. See notes ^6^ for more information.
    Some reasoning ^8^ suggests that a minor allele frequency threshold should be set to 10/n where n is the number of markers. The #MAF parameter can be set to ‘na’ which will use 10/n as a threshold or a threshold value can be explicitly given, such as ‘.01’.
3. Heterozygosity
    Individuals resulting from random mating within a population should have predictable heterozygosity (H) values. H is a measure of the number of loci in an individual that are heterozygous. Departure from expected H values can signify DNA quality issues (high H) or samples from a different population (low H). This step can be turned off by not supplying the ‘#HET’ parameter in the parameter file. As long as the parameter is listed, this step will be done. H and the inversely related F (Method-of moments F coefficient estimate) are investigated using PLINK ‘--het’. F is calculated as the ([observed homozygous count] - [expected count])/([total observations] - [expected count])) where the expected count is calculated from an imputed MAF. A histogram of F values is generated for manual investigation.
    If values for ‘#FMIN’ and ‘#FMAX’ are supplied in the parameter file then samples with an F value below ‘#FMIN’ and above ‘#FMAX’ are removed. If these values are not supplied then samples above or below three standard deviations of the mean H are removed, as suggested be Anderson *et al.* ^1^.
4. Hardy-Weinberg equilibrium (HWE)
    Markers out of HWE can indicate that there were genotyping errors. However, a strong association signal can also result in deviations from HWE. So here only variants from control samples are checked for deviations from HWE. PLINK ‘--hardy’ is used to generate HWE p-values and a Q-Q plot of the log-P-values of the markers for the controls is generated for manual investigation. A p-value threshold is supplied in the parameter file to remove markers with a p-value lower than expected.
5. Cryptic relatedness
    Cryptic relatedness (CR) is when pairs of individuals are closely related and can lead to false positive or negative correlations when subjects are treated as independent. The PLINK ‘--genome’ command can estimate relatedness, but is quite slow when there are a large number of markers in a dataset. Therefore, markers in high linkage disequilibrium (LD) are removed first to thin the data. This is done using PLINK ‘--indep-pairwise’ with parameters suggested by Weale ^6^. This creates a pruned data set that contains markers with a minimal LD (which is caused by limited recombination occurring between two or more loci and results in a non-random association between the loci). Furthermore, only assayed markers are used in this step (i.e. not imputed markers). Using a pruned data set is advantages because CR methods work best when no LD is assumed between markers and it also reduces the input size and in turn greatly reduces the computation time.
    PLINK ‘--genome’ estimates relatedness of all pairs of samples and reports identify by decent (IBD, a measure of whether identical regions of two genomes were inherited from the same ancestry) in the PI_HAT (actually, proportional IBD, i.e. P(IBD=2) + 0.5*P(IBD=1)) column of the result file. A PI_HAT value close to 1 would indicate a duplicate sample. The threshold 0.1875 represents the half-way point between 2nd and 3rd degree relatives and is a common cut-off to use. Of each pair of related individuals, the one with the greater proportion of missing SNPs is dropped from the final dataset.
6. Principal component analysis (PCA)
    Generally, PCA transforms a data matrix (such as a GWAS n x m matrix where n in is the number of individuals and m is the number of markers and each element in the matrix represents the scaled genotype for the particular individual at that particular marker) so that the successive principal components are not correlated. The number of PCs is less than or at most equal to the original number of columns and the first PC explains the largest variance in the genotype data. Traditionally, PCA is used to (1) screen the study population for heterogeneous ethnic backgrounds and (2) to correct for potential population stratification (the difference of allele frequencies in ancestral subpopulations). It can be seen in Figure 2 where HapMap ^9, 10^ data with individuals with known ancestry are included in the PCA, when plotting the first two PCs subpopulations cluster together. HapMap is an international project that aims to identify genetic similarities and differences between populations.
    As with the cryptic relatedness step, a thinned dataset created with PLINK and starting from assayed markers only is used to calculate PCs. The SMARTPCA tool is used to calculate PCs from this thinned dataset and identify outliers for removal. The PCs can then be used for further corrections in analysis models.

*Data Formats.* The input GWAS data are expected to be in PLINK bfile format. The input data will have three files associated to it with .bed, .bim, and .fam file extensions. The .bed file is a binary file that contains the genotype information for all individuals ( https://www.cog-genomics.org/plink2/formats#bed). The .bim file is a mapping file giving information on each marker ( https://www.cog-genomics.org/plink2/formats#bim). The .fam file gives information on each individual ( https://www.cog-genomics.org/plink2/formats#fam).

This pipeline utilizes information that was not provided in the original PLINK files and therefore the phenotype is always provided in an alternate phenotype file. PLINK ‘–pheno’ is used to provide the phenotype file and PLINK ‘–pheno-name’ is used to provide the phenotype name which also corresponds to the header of the column in the phenotype file. The first two columns in the phenotype file must have the column headers ‘FID’ and ‘IID’ respectively. ‘FID’ is the family ID or ‘0’ if not used and ‘IID’ is the individual ID that corresponds to the ‘IID’ values in the .fam file ( https://www.cog-genomics.org/plink2/input#pheno).

### Operation

*System.* The pipeline was tested on STATGEN, a Dell PowerEdge R520 server with two Intel Xeon E5-2470 CPUs (32 cores at 2.3GHz), 24TB of storage in a RAID6 array with two drive fault tolerance, and 128GB of RAM. The operating system is Ubuntu Server 14.04 LTS 64-bit edition.

*Required software.* The automated pipeline is written in Python and calls Rscript, PLINK, and SMARTPCA. The versions used for building and testing are the following,

1. Python - Python 2.7.6
2. Rscript - R scripting front-end version 3.2.2
3. PLINK - PLINK v1.90b3x 64-bit
4. SMARTPCA – smartpca version 13050

**Figure 3.**
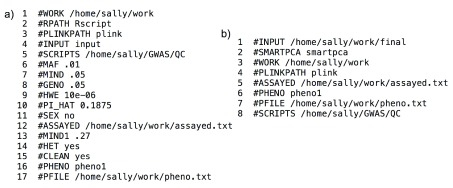
Parameter files **a**) qc_params.txt **b**) pca_params.txt.

*Parameter files.* Example parameter files are shown in Figure 3. The QC.py and PCA.py scripts read in parameters and names them based on the word following the ‘#’ and gives that parameter the value following the white space on the same line. The line numbers in the figure are not a part of the parameter file (i.e. each line starts with ‘#’). The parameters and values are stored in a python dictionary, so order and extra parameters do not matter. However, the exact name and case of the parameters are important for the scripts to correctly function. The parameters described here are ordered by the line number given in the qc_params.txt file in Figure 3a. The parameters with the same name in the pca_params.txt file in Figure 3b have the same meaning.

  1. WORK – path to the working directory where all generated files will be written
  2. RPATH – path to where Rscript is located, or name if in a known path
  3. PLINKPATH – path to the PLINK executable, or name if in a known path
  4. INPUT – input file in PLINK bfile format
  5. SCRIPTS – path to directory in which the helper scripts called by the pipeline live
  6. MAF – minor allele frequency threshold
  7. MIND – individual missingness threshold
  8. GENO – marker missingness threshold
  9. HWE – Hardy-Weinberg threshold
10. PI_HAT – IDB threshold
11. SEX – if value is equal to ‘yes’ then a PLINK sex check is performed, otherwise it is not
12. ASSAYED – location of file that includes names of all assayed markers in a study to ensure that imputed SNPs are not used for some QC steps
13. MIND1 – an optional, first tier (non-stringent) individual missingness threshold
14. HET – if this parameter is included then the Heterozygosity step is performed (NOTE since ‘#FMIN’ and ‘#FMAX’ are not given, this parameter file will result in the removal of markers with an H value +/- 3 standard deviations)
15. CLEAN – will result in the removal of intermediate files and final PLINK finals to be named ‘final’
16. PHENO – the name of the phenotype that will be investigated during analysis. Some QC steps vary based on whether or not an individual is case, control, or undefined.
17. PFILE – the files in which the phenotype is given formatted in the proper PLINK format with FID, IID, pheno1 column headers where FID is the family ID (commonly 0 if relations not known), the individual’s ID, and the phenotype value encoded as 1=unaffected (control) and 2=affected (case).
18. SMARTPCA (line #2 in pca_params.txt and not in qc_params.txt) – link to the smartpca executable.

*Running scripts and results*

1. QC.py
    Once the parameters and values are correctly written to the parameter file, the script is executed by calling the parameter file as a command line argument as follows,
                                                      python QC.py qc_params.txt
The QC steps are performed as described in the Implementation section and after each step a count is retrieved for the number of individuals or markers removed in each step. A log file is written (final_QC.log). All of the parameters from qc_params.txt are first written to the log file to ensure the process can be duplicated. Then the number of individuals/markers removed during each step are recorded along with the running total of how many individuals and markers are left in the dataset. This allows for easy comparison of the overall effects of different parameter settings. If ‘#CLEAN’ is set then the final QC dataset is in PLINK format and named ‘final.bed’, ‘final.bim’, and ‘final.fam’. There will be an archived file ‘final_graphs.tgz’ containing the files missing.png, het.png, hwe.png, and relate.png, created in the thresholds, hetereozygosity, and HWE steps above for manual inspection in case parameters need to be adjusted and QC redone.
2. PCA.py
    Once the parameters and values are correctly written to the parameter file, the scripts is executed by calling the parameter file as a command line argument as follows,
                                                      python PCA.py pca_params.txt
    The data thinning is carried out in PLINK, the input file for SMARTPCA is automatically generated, and then SMARTPCA is called to calculate the PCs. If the analysis is being done in R, then the ‘smartpca.evec’ file can be read in and merged to exclude individuals in which PCs were not calculated (i.e. outliers). PCA.py also generates a file called ‘remove.txt’ in PLINK format with an FID (all marked ‘0’) and IID column so that the outliers can be removed during PLINK analysis with the ‘–remove’ command.

## Conclusions

While the QC steps given here are not novel, this paper provides access to an automated process that both reduces human work time and chances for error and provides tools to make the computational experiment reproducible. It also gives recommended values for parameters but facilitates changing parameters and the comparison of effects.

## Software availability

Zenodo: GWAS: Automated GWAS QC, doi: 10.5281/zenodo.58228 ^11^.

GitHub: https://github.com/sallyrose0425/GWAS, https://github.com/sallyrose0425/GWAS/blob/master/LICENSE
