# Supplementary material for: Automated quality control for genome wide association studies
Source: F1000Res. 2016 Jul 29;5:1889. [Version 1] doi: 10.12688/f1000research.9271.1 (PMC5007749; doi:10.12688/f1000research.9271.1)
Supplement: Peer review history

# Peer review: Referee response for version 1

**DOI:** 10.5256/f1000research.9982.r15446

**Published:** 2016-08-25

**Authors:** Brad Chapman

**Affiliations:** 1 Department of Biostatistics, Harvard Public School of Health, Boston, MA, USA

The authors describe a useful tool for performing automated quality control analysis of GWAS input data. They provide a set of script written in Python and R that automate running plink and smartpca to check for common issues with input data. This is a valuable contribution for documenting and automating the processes involved with checking GWAS input data.

 I have some suggestions for improving the usefulness of the tool:

- A description of the input data would help users get started with this tool. Does it take only array genotyping data or also sequencing data? Is different pre-processing or QC threshold adjustment required for the different types of data?
- The software needs a minimal set of example files that users can run to confirm they have the necessary system tools installed. This will help distinguish errors in installation/running from errors in the input data. It will also help users understand the exact inputs, including plink phenotype files. It's quite hard to get started with any tool without test data. If you run into issues it is hard to distinguish differences/problems with your dataset from issues with the code.
- It would be helpful for tool usage if it had a unique name. Naming the scripts generically as GWAS (or GWAS QC) makes it hard to reference or find information about the tool.

I have read this submission. I believe that I have an appropriate level of expertise to confirm that it is of an acceptable scientific standard, however I have significant reservations, as outlined above.

# Peer review: Referee response for version 1

**DOI:** 10.5256/f1000research.9982.r15797

**Published:** 2016-08-22

**Authors:** Hilary Coller

**Affiliations:** 1 Department of Molecular, Cell and Developmental Biology, University of California Los Angeles, Los Angeles, CA, USA

**Title and abstract:** The title and abstract are appropriate and suitably summarize the work.

**Article content:** The article describes a series of scripts that the authors have developed to provide quality control measures for genomewide association studies The scripts address critical questions for genomewide association study datasets such as checking the extent of heterozygosity to determine whether it is as expected, checking whether there are markers that do not follow Hardy-Weinberg equilibrium predictions, and checking whether there are pairs of closely related individuals. The authors provide a helpful series of scripts that will allow users to perform these tasks in a reproducible and automated fashion. The scripts make it easy to determine the number of samples removed for different parameter settings. These scripts are likely to be valuable for researchers in the field.

**Conclusions:** The conclusions of the manuscript are appropriate, that use of these scripts will allow for consistent quality control of genomewide association study datasets, which will lead to better interpretation of the available data.

I have read this submission. I believe that I have an appropriate level of expertise to confirm that it is of an acceptable scientific standard.

# Peer review: Referee response for version 1

**DOI:** 10.5256/f1000research.9982.r15320

**Published:** 2016-08-09

**Authors:** Prasoon Agarwal

**Affiliations:** 1 Division of Clinical Immunology, Department of Laboratory Medicine, Karolinska Institutet, Huddinge, Sweden

The current manuscript by Ellingson *et al., *is regarding the automation of the quality control (QC) of genome wide association studies (GWAS). Automation is important as it reduces human errors and increases work efficiency. In this manuscript authors have written a python script that automates the process of QC of GWAS data. The QC steps in this pipeline follow the notes developed by MikeWeale *et al.*

 However, there are some issues with the current version of the manuscript.

1. There is/are already software like QCGWAS ^1^, which are automated for QC of GWAS. So how is the current version of the software different or better from the already existing softwares. So authors should try to summarize by comparing in terms of efficiency, accuracy in performance of their work with the already present softwares in this field.
2. Authors should provide a link to a test dataset that can be used to check if the required softwares are installed and running properly.
3. The introduction should be carefully written citing the known automated softwares for GWAS. 

Overall the current version of manuscript the authors have failed to bring the uniqueness of their work in the field .

I have read this submission. I believe that I have an appropriate level of expertise to confirm that it is of an acceptable scientific standard, however I have significant reservations, as outlined above.

## References

[1] van der MostPJVaezAPrinsBPMunozMLSniederHAlizadehBZNolteIM: QCGWAS: A flexible R package for automated quality control of genome-wide association results. *Bioinformatics*.2014; 10.1093/bioinformatics/btt745 10.1093/bioinformatics/btt745 24395754
